# Growth, physiological, and molecular responses of three phaeophyte extracts on salt-stressed pea (*Pisum sativum* L.) seedlings

**DOI:** 10.1186/s43141-023-00483-z

**Published:** 2023-03-16

**Authors:** Marwa M. Hamouda, Abdelfattah Badr, Sameh S. Ali, Alia M. Adham, Hanan I. Sayed Ahmed, Khalil M. Saad-Allah

**Affiliations:** 1 Botany Department, Faculty of Science, Tanta University, Tanta, 31527 Egypt; 2 Botany and Microbiology Department, Faculty of Science, Helwan University, Cairo, 117900 Egypt; 3 Plant Protection Department, Faculty of Agriculture, University of AL-Muthanna, Samawah, Iraq

**Keywords:** Seaweeds, Pea, Salinity, Osmoregulation, ISSR fingerprinting

## Abstract

**Background:**

Seaweeds are a viable bioresource for suffering plants against salt stress, as they abundant in nutrients, hormones, vitamins, secondary metabolites, and many other phytochemicals that sustain plants' growth under both typical and stressful situations. The alleviating capacity of extracts from three brown algae (*Sargassum vulgare*, *Colpomenia sinuosa*, and *Pandia pavonica*) in pea (*Pisum sativum* L.) was investigated in this study.

**Methods:**

Pea seeds were primed for 2 h either with seaweed extracts (SWEs) or distilled water. Seeds were then subjected to salinity levels of 0.0, 50, 100, and 150 mM NaCl. On the 21st day, seedlings were harvested for growth, physiological and molecular investigations.

**Results:**

SWEs helped reduce the adverse effects of salinity on pea, with *S. vulgare* extract being the most effective. Furthermore, SWEs diminished the effect of NaCl-salinity on germination, growth rate, and pigment content and raised the osmolytes proline and glycine betaine levels. On the molecular level, two low-molecular-weight proteins were newly synthesized by the NaCl treatments and three by priming pea seeds with SWEs. The number of inter-simple sequence repeats (ISSR) markers increased from 20 in the control to 36 in 150 mM NaCl-treated seedlings, including four unique markers. Priming with SWEs triggered more markers than the control, however about ten of the salinity-induced markers were not detected following seed priming before NaCl treatments. By priming with SWEs, seven unique markers were elicited.

**Conclusion:**

All in all, priming with SWEs alleviated salinity stress on pea seedlings. Salinity-responsive proteins and ISSR markers are produced in response to salt stress and priming with SWEs.

**Supplementary Information:**

The online version contains supplementary material available at 10.1186/s43141-023-00483-z.

## Background

One of the most significant abiotic stress factors affecting agriculture globally is salinity [1]. Plants are harmed by salinity because it alters their metabolism, slows their growth, and produces toxicity [2]. Salinity causes wilting, drying, and death of entire organs by interfering with photosynthesis, respiration, and assimilate distribution [3]. Salinity also reduces the quality and productivity of crop plants and results in a monetary loss in agricultural production [4]. Salt stress has multi-factorial; particularly, a high salt content creates osmotic stress, which reduces water absorption by the roots and induces the production of reactive oxygen species (ROS), which affects plant growth and development, as well as physiological and metabolic activities [5, 6]. Salt tolerance, on the other hand, is a complicated process that involves physiological, biochemical, and molecular mechanisms [6, 7]. Sofy et al. [8] assumed an association of antioxidant defenses with long-term salt stress in pea.

Pea (*Pisum sativum* L.) is a prominent legume that has been grown in many parts of the world for centuries. Pea seeds are a good source of protein, carbohydrates, fibers, vitamins, and minerals [9]. Pea seed protein is easy to digest and contains a well-balanced amino-acid composition [10]. Pea is grown in temperate regions of the world as well as a winter crop in the subtropics for fresh green seeds, tender green pods, dried seeds, and foliage [11]. The pea plant has a shorter growing season than many other broad-leaved crops and demands less water [12]. Pea yield has been reported to be lowered by 50% when exposed to moderate salinity (100 mM NaCl) [13], but when exposed to extreme salinity, its yield is drastically reduced [14].

Seaweeds have been used as animal feed and as food in several cultures due to their high content of protein, fiber, polyunsaturated fat, and minerals [15]. However, heavy metals in seaweeds constitute a potential risk to human health [16]. Seaweeds may be used for producing natural plant growth stimulants to improve plant physiological responses in salt-stressed conditions. Seaweed extracts exhibit plant growth regulators such as indole acetic acid (IAA), gibberellins, cytokinins, choline chloride, and glycine betaine, which are involved in a variety of physiological processes in plants and have an amenable impact on crop quantity and quality [17, 18]. Seaweed extracts were also employed to produce biofertilizers [19]. The priming of plant seeds with seaweed extracts improved germination in a range of species. This is supported by the studies made on wheat [18, 20], green gram [21], common bean [22], barley [23], radish [24], and pepper [25]. Furthermore, Ahmed et al. [26] reported increased radish growth in soil polluted with heavy metals (Pb, Cu, Zn, and Ni) mixed with dried seaweeds as a result of nutrients and growth hormones present in the seaweeds, and concluded that seaweeds could be used in eco-friendly soil pollution bioremediation.

Seed priming is one of the most appealing, low-cost, and low-risk technologies for improving germination and plant growth [18]. By altering seed vigor and/or physiological status, it helps to improve seed germination and seedling establishment [2, 24]. The primed seeds germinate and establish seedlings faster, more uniformly, and more successfully [18]. In several crops, priming improved germination rate, field emergence, seedling vigor, stand establishment, and economic yield [27, 28], as well as promoted biotic and abiotic stress resistance [18, 29]. Extracts from the green algae *Ulva fasciata*, *Cystoseira compressa*, and *Laurencia obtusa* greatly improved seed germination and seedling growth in *Vigna sinensis* and *Zea mays*, according to [30].

SDS-PAGE pattern variation has been commonly used as a biochemical marker in plant species systematics and crop breeding, as well as in plant response to environmental stress, particularly salinity and water stress [31, 32]. SDS-PAGE pattern variation has also been documented as a result of employing seaweed liquid fertilizers on some crops in several investigations [24, 33, 34]. DNA Inter-Simple-Sequence Repeats (ISSRs) fingerprinting polymorphism has been employed as a molecular marker to determine genetic variation among agricultural plant populations [35], as well as for differentiating closely related genotypes and cultivar identification in plant species [36, 37]. Furthermore, ISSR fingerprinting has been used to distinguish genotypes and populations [3832]. ISSR was utilized to detect genetic divergence among different barley genotypes under salt stress [39]. It was also used in conjunction with biochemical markers to identify the divergence of wheat genotypes [40]. Accordingly, the main objective of this study is to see how extracts from three brown macroalgal species (*Colpomenia sinuosa*, *Padina pavonica*, and *Sargassum vulgare*) influence pea (*Pisum sativum* L.) seed germination and seedling growth, as well as their effect on protein SDS-PAGE patterns and ISSR DNA fingerprinting of salt-stressed pea seedlings.

## Methods

### Collection of seaweeds and preparation of their extracts

In this study, three brown algae species from the Phaeophyceae family were chosen, these are *Colpomenia sinuosa* Martens ex Roth, *Pandia pavonica* Thivy, and *Sargassum vulgare* Agardh. Fresh materials of these seaweeds were collected from Rocky Bay of Abu Qir in Alexandria, Egypt (longitudes 30°05′–30°22′ E and latitudes 31°16′–31°21′ N). These species were identified using Aleem [41] as a guide and authenticated using the algal Base website [42]. Seaweed samples were cleaned and oven-dried at 60 °C for 72 h. The dried materials were ground to a fine powder in an electric mill, and the water extract of milled material was obtained at 10 g/L water with continuous shaking for 24 h. After that, the extracts were filtered through Whatman No. 1 filter paper and kept at 4 °C until utilized.

### Pea seeds priming with seaweed extracts and NaCl treatments

Agricultural Research Center, Giza, Egypt, generously provided seeds of pea (*Pisum sativum* L.) cultivar “Master B”. Seeds were surface-sterilized for with a 5% Clorox solution, then rinsed three times with tap water and once with distilled water. Sterilized pea seeds were immersed in either *C. sinuosa, P. pavonica*, or *S. vulgare* extracts or distilled water for 2 h, then completely rinsed with distilled water. All soaking seeds were planted in plastic pots with a diameter of 40 cm and a depth of 20 cm, filled with 8 kg of clay-sandy soil (2:1w/w), and watered with tap water. To calculate the final germination percentage, daily seed germination was recorded for 5 days. After 5 days of planting, the pots of each priming treatment were watered with 50,100, or 150 mM NaCl solutions, whilst the pots of the control samples were irrigated with tap water. Every week, the pots were given solutions with 80% of their field capacity. The physio-chemical properties of the soil used in this experiment, as detected according to the procedures described by Alvarenga et al. [43], showed that it has a clay loamy texture, a pH of 7.71, an EC of 1.62 dS.m^−1^, and a bulk density of 1.31 g.cm^3^. This soil contained total nitrogen (1.04), phosphorus (0.35), potassium (3.48), magnesium (5.32), sodium (3.84), chloride (4.82), total carbonate (3.37), and active carbonate (1.48) mg.kg^1^ soil, according to the mineral content analysis.

### Evaluation of growth parameters

When the radical had protruded through the seed coat and was at least 2 mm long, seeds were considered germinated. On the 21st day of germination, the root and shoot lengths of five pea seedlings were measured (cm) using a ruler and weighed to calculate their fresh weight (g).

### Physiological traits

The photosynthetic pigments of pea seedlings were extracted in cold 80% acetone and evaluated as mg g^−1^ FM utilizing methods outlined by Metzner et al. [44]. The acid ninhydrin reagent and a standard curve prepared by proline were used to quantify the content of proline as anti-stress amino acid in the leaf powders of pea seedlings as outlined by Bates et al. [45]. The osmoregulatory molecule glycinebetaine (GB) was measured in the aqueous leaf extract of pea leaves dry powder using the approach of Grieve and Grattan [46] and a standard graph established by GB. Proline and GB contents were reported in mg g^−1^ DM.

### Molecular markers

#### Protein SDS-PAGE

The total protein of both independent and combined treatments was extracted from germinating pea seedlings using 30 mM Tris–HCl buffer (pH 8.0) supplemented with 0.2% SDS, 3 M urea, and 1 ml of β-mercaptoethanol. Seedling proteins were separated in a 12.5% polyacrylamide gel using SDS-PAGE type. The electrophoresis was carried out at 100 V and 15 mA using a power supply (DYY-12 power supply Beijing Liuyi, Germany). The molecular weight marker (5 ll) made up of 10–200 kDa proteins (Fermentas, Germany) was applied as a standard marker in parallel with the samples. After electrophoresis, the Coomassie Brilliant BlueR-250 stain was used to stain the protein bands on the gel, which was then removed overnight with a mixture of methanol and acetic acid. The gel was then photographed with a 9.2-megapixel Kodak digital camera Model AF3X optical aspheric lens. The molecular weights of the protein bands were determined using Kapelan GmbH’s Lab Image software version 2.7.

### ISSR amplification and electrophoresis

Total genomic DNA was extracted from 100 mg of young pea young leaves using the Gene JET™ Plant Genomic DNA Purification Kit (K0721/Thermo Fisher, UK) according to the instructions of the manufacturer. The ISSR fingerprinting protocol was based on the approach described by Badr et al. [38]. A total of 10 ISSR primers (Operon Nippon EGT CO. LTD) were used and only 7 primers produced a clear readable profile. Table 1 lists the primer sequences as well as the annealing temperatures. The PCR amplification was carried out on a Bio-Rad thermo-cycler using the following cycle profile: initial denaturation at 95 °C for 8 min, followed by 40 cycles of 30 s at 94 °C, 30 s at 60 °C, and 30 s at 72 °C, and 5 min at 72 °C for final product extension. Electrophoresis of the amplified PCR products was done in a 1.8% agarose gel containing ethidium bromide (0.5 µg/ml) in 1TAE buffer at 100 V to resolve the amplified PCR products. The ISSR fingerprinting was seen and photographed under UV light using a Gel Works 1D advanced gel documentation system (UVP, UK). The size of each band was determined using a standard marker of 100 bp DNA ladder (Fermentas, Germany) and Kapelan GmbH’s Lab Image software (version 2.7).

**Table 1:** List of ISSR primers utilized in this study, their sequences, annealing temperatures, number of bands produced by each primer, and polymorphism percentage

#	Primer name	Sequence (5′-3′)	Annealing temperature (ºC)	Primer length (bp)	Number of bands	Band type			Polymorphism (%)
						*P*	*M*	*U*	
1	808	AGAGAGAGAGAGAGAGC	59	17	5	3	1	1	60.0%
2	827	ACACACACACACACACG	62	17	6	3	2	1	50.0%
3	HB-15	GTG GTGGTG GC	57	11	8	1	4	3	12.5%
4	829	TGTGTGTGTGTGTGTGC	62	17	10	6	2	2	60.0%
5	HB-8	GAGAGAGAGAGAGG	44	14	5	3	2	0	60.0%
6	CAG	CAGCAGCAGCAGCAGCAG	56	18	6	4	1	1	66.6%
7	17898B	CACACACACACAGT	42	14	11	5	3	3	45.5%
	Total				51	25	15	11	

*P* Polymorphic, *M* Monomorphic, *U* Unique

### Cluster analysis of protein and ISSR data

Based on variance in SDS-PAGE protein profiles and ISSR fingerprinting of seven primers, cluster analysis was utilized to represent the similarity of pea seedlings following seeds priming with seaweed extracts, NaCl treatments, and their combined interactions (Supplementary Tables S1 and S2). In binary matrices for cluster analysis, the presence and absence of protein and ISSR bands on the gel were scored as (1) for presence and (0) for absence. The binary matrices for cluster analysis were produced depending on the distance between control seedlings and the other 15 treatments based on protein SDS-PAGE and ISSR fingerprinting. The binary data of pea 16 treatments were calculated using the Community Analysis Package-5 (CAP) [47] to create an average linkage distance tree based on the hierarchical grouping function and the PAST software (V.3.22) with the aid of the Paleontological Statistics software developed by Hammer et al. [48] to create a distance tree based on the Dice’s similarity coefficient. The two cluster analyses were performed using the Unweighted Pair Group Method with Arithmetic Mean (UPGMA) algorithm as implemented in the CAP and PAST software.

### Statistical analysis

Measurements were made in triplicates for all the examined parameters and the data were expressed as the mean ± standard deviation (SD) of three separate replications. The two-way analysis of variance (ANOVA) was used to assess the effect of different salt concentrations and the three types of seaweed extracts, as well as their combined interactions on the growth parameters and physiological traits. A one-way ANOVA was also conducted to establish the degree of significance across different treatments and to separate means using Tukey’s HSD test at a 5% significance level. Minitab 19 software was used to conduct all of the analyses.

## Results

### Growth performance

Table 2 depicts how the pea plant responds to different NaCl concentrations (0.0, 50, 100, and 150 mM), three seaweed extracts (*Sargassum vulgare*, *Pandia pavonica*, and *Colpomenia sinuosa*), and their combined interactions in terms of germination rate, seedling length, and seedling fresh weight. Following treatment with NaCl concentrations of 50, 100, and 150 mM, the germination percentage decreased from 84.53% in control plants to 73.67, 66.93, and 63.60%, respectively. The seaweed extract (SWE) priming treatments, on the other hand, considerably boosted the germination percentage compared to the control, with a germination percentage of 92.80% for the *S. vulgare* extract, 89.07% for *P. povonica*, and 85.87% for *C. sinuosa*. In comparison to salt treatments individually, the three algal extracts boosted the germination rate in all salt-stressed pea seedlings, particularly the combined treatment of 50 mM NaCl and *S. vulgare* extract.

**Table 2:** Germination percentage, seedling length, and fresh weight of 21-day-old pea seedlings treated with NaCl and SWEs (*S. vulgare*, *C. sinuosa*, and *P. pavonica*) single and combined treatments

Treatments	Germination rate (%)	Shoot length (cm)	Root length (cm)	Shoot fresh weight (g)	Root fresh weight (g)
Control	84.53 ± 0.46^ cd^	14.1 ± 0.36^ab^	19.3 ± 0.95^bc^	1.35 ± 0.009^b^	2.34 ± 0.053^bc^
50 mM NaCl	73.67 ± 0.81^f^	11.3 ± 0.26^def^	16.2 ± 0.15^ef^	0.86 ± 0.015^f^	1.93 ± 0.011^ef^
100 mM NaCl	66.93 ± 1.66^ h^	10.6 ± 0.25^efg^	15.6 ± 0.351^ fg^	0.54 ± 0.002^ij^	1.08 ± 0.002^ k^
150 mM NaCl	63.60 ± 0.40^i^	8.7 ± 0.62^ h^	13.8 ± 0.26^ h^	0.40 ± 0.001^ l^	0.84 ± 0.001^ m^
*Sargussum vulgare*	92.80 ± 0.80^a^	15.5 ± 0.50^a^	21.0 ± 0.10^a^	1.47 ± 0.011^a^	2.64 ± 0.005^a^
* S. vulgare* + 50 mM NaCl	81.60 ± 0.80^de^	13.8 ± 0.26^bc^	17.6 ± 0.38^de^	1.06 ± 0.002^d^	2.06 ± 0.001^d^
* S. vulgare* + 100 mM NaCl	74.07 ± 1.14^f^	13.8 ± 1.06^bc^	17.8 ± 0.21^ cd^	0.72 ± 0.008^ h^	1.78 ± 0.015^ g^
* S. vulgare* + 150 mM NaCl	71.40 ± 0.87^ fg^	10.3 ± 0.35^ fg^	14.4 ± 0.56^gh^	0.95 ± 0.010^e^	1.56 ± 0.003^ h^
*Colpomenia sinuosa*	85.87 ± 1.22^c^	14.9 ± 0.324^ab^	20.7 ± 0.29^ab^	1.16 ± 0.038^c^	2.26 ± 0.020^c^
* C. sinuosa* + 50 mM NaCl	73.67 ± 0.81^f^	11.8 ± 0.21^de^	17.6 ± 0.55^de^	0.82 ± 0.008^ fg^	1.98 ± 0.015^de^
* C. sinuosa* + 100 mM NaCl	72.80 ± 0.80^f^	12.3 ± 0.416^d^	17.8 ± 0.568^ cd^	0.56 ± 0.017^i^	1.43 ± 0.020^i^
* C. sinuosa* + 150 mM NaCl	68.53 ± 1.22^gh^	9.6 ± 0.60^gh^	13.0 ± 0.057^ h^	0.48 ± 0.004^ k^	1.02 ± 0.014^kl^
*Pandia pavonica*	89.07 ± 1.22^b^	14.6 ± 0.35^ab^	19.5 ± 0.65^ab^	1.42 ± 0.010^a^	2.41 ± 0.086^b^
* P. pavonica* + 50 mM NaCl	79.47 ± 0.46^e^	12.5 ± 0.40^ cd^	17.2 ± 0.15^de^	0.79 ± 0.051^ g^	1.84 ± 0.047^ fg^
* P. pavonica* + 100 mM NaCl	72.00 ± 1.20^f^	12.1 ± 0.15^d^	16.6 ± 0.49^def^	0.52 ± 0.006^ijk^	1.31 ± 0.055^j^
* P. pavonica* + 150 mM NaCl	65.87 ± 1.41^hi^	9.6 ± 0.53^gh^	15.4 ± 0.46^ fg^	0.48 ± 0.003^jk^	0.94 ± 0.006^ l^
Significance					
Salinity (S)	938.08 ***	254.73 ***	269.62 ***	4881.14 ***	3831.59 ***
Seaweed extracts (SWEs)	121.06 ***	44.58 ***	18.67 ***	685.65 ***	463.56 ***
(S × SWEs)	9.19 ***	2.26 *	6.66 ***	94.08 ***	52.13 ***

The values shown are average of three replicates ± standard deviation

Numbers represent the *F* value at 5% significance level

^*^P ˂ 0.05

^**^P ˂ 0.01

^***^P ˂ 0.001

When compared to the control, all NaCl-induced salinity treatments resulted in a concentration-dependent reduction in shoot and root length of the 21-day-old pea seedlings. Seed priming with the three SWEs resulted in a relative increase in pea shoot and root lengths. In comparison to other treatments, *S. vulgare* produced the longest shoots (15.5 cm) and roots (21.0 cm). In comparison to the non-primed salt-stressed controls, when seaweed treatments were combined with salt stress, the inhibitory effect of NaCl on the shoot and root lengths was diminished (Table 2).

In comparison to the corresponding controls, there was a substantial reduction in the fresh weight of the 21-day-old pea seedlings shoot and root as the NaCl concentration increased. When compared to the control fresh weight, the highest NaCl dosage (150 mM) resulted in a 70.4% decrease in shoot fresh weight and a 64.0% decrease in root fresh weight. When compared to control weights, seed priming with *S. vulgare* and *P. pavonica* extracts enhanced seedling shoot and root fresh weights, but seed priming with *C. sinusa* extract decreased them. Nonetheless, the combination of NaCl and priming treatments had a varied effect on the shoot and root fresh weights of pea seedlings, with *S. vulgare* extract being the most effective in reducing salt deterioration on the shoot and root fresh weights.

In this work, the statistical analysis (two-way ANOVA) of the growth performance attributes revealed that all experimental treatments, including salinity, SWEs, and their combined interactions, had a significant (*P* ˂ 0.05) impact on all of the assessed growth traits.

### Physiological responses

 The results of the present work demonstrated that photosynthetic pigments, proline, and glycinebetaine (GB) of pea seedlings all were affected by salt treatments, and SWEs priming as well as their combined interactions (Table 3). Salt stress significantly reduced Chl a and Chl b levels, with the highest salt concentration (150 mM) causing 48.72 and 32.67% declines in Chl a and Chl b, respectively, when compared to the equivalent controls. Priming with SWEs significantly improved the amount of chlorophyll in pea leaves, with *S. vulgare* being the most relevant for Chl a and *C. sinousa* for Chl b. The use of SEWs in combination with salinity treatments reduced the chlorophyll content loss caused by salt stress. *P. pavonica* was the best for Chl a and *C. sinousa* was best for Chl b at the highest salt concentration. Carotenoids, on the other hand, were found in higher concentrations in salt-stressed pea leaves. When compared to the counterpart control, the highest salt treatment resulted in a 58.10% rise in carotenoids. Interestingly, SWEs priming resulted in a higher accumulation of carotenoids in comparison to the un-stressed control plants. The most detected rise in carotenoids following SWEs priming was recorded after *P. pavonica*, which prompted a 61.16% increment above the control content.

**Table 3:** Physiological traits (Chl a, Chl b, carotenoids, proline, and glycinebetaine) of 21-day-old pea seedlings treated with NaCl and SWEs (*S. vulgare*, *C. sinuosa*, and *P. pavonica*) single and combined treatments. The values shown are average of three replicates ± standard deviation

Treatments	Chl a (mg g^−1^ FM)	Chl b (mg g^−1^ FM)	Carotenoids (mg g^−1^ FM)	Proline (mg g^−1^ DM)	Glycinebetaine (mg g^−1^ DM)
Control	10.53 ± 0.25^c^	4.50 ± 0.26^bc^	3.27 ± 0.32^ h^	6.83 ± 0.15^ef^	1.80 ± 0.19^ef^
50 mM NaCl	8.30 ± 0.36^f^	3.70 ± 0.17^def^	3.43 ± 0.25^gh^	7.67 ± 0.21^ cd^	2.33 ± 0.21^c^
100 mM NaCl	6.57 ± 0.21^ h^	3.33 ± 0.21^ fg^	4.53 ± 0.21^ cd^	8.73 ± 0.25^b^	3.19 ± 0.18^ab^
150 mM NaCl	5.40 ± 0.10^i^	3.03 ± 0.32^ g^	5.17 ± 0.25^ab^	10.77 ± 0.21^a^	3.55 ± 0.13^a^
*Sargussum vulgare*	11.60 ± 0.20^a^	4.83 ± 0.32^ab^	4.27 ± 0.40^de^	5.87 ± 0.15^ g^	1.83 ± 0.26^def^
* S. vulgare* + 50 mM NaCl	9.83 ± 0.15^d^	4.17 ± 0.35^ cd^	3.77 ± 0.31^efgh^	6.55 ± 0.16^ef^	2.26 ± 0.29^c^
* S. vulgare* + 100 mM NaCl	8.13 ± 0.31^f^	4.00 ± 0.26^de^	4.60 ± 0.10^ cd^	7.70 ± 0.36^c^	2.91 ± 0.20^b^
* S. vulgare* + 150 mM NaCl	6.53 ± 0.15^j^	3.33 ± 0.21^ fg^	4.44 ± 0.20^d^	9.07 ± 0.38^b^	3.04 ± 0.32^b^
*Colpomenia sinuosa*	11.10 ± 0.30^b^	5.27 ± 0.15^a^	4.23 ± 0.40^def^	6.53 ± 0.42^ef^	1.73 ± 0.08^f^
* C. sinuosa* + 50 mM NaCl	9.33 ± 0.21^e^	4.10 ± 0.26^ cd^	3.73 ± 0.21^fgh^	6.64 ± 0.39^ef^	2.10 ± 0.21^cde^
* C. sinuosa* + 100 mM NaCl	7.40 ± 0.20^ g^	3.80 ± 0.26^def^	4.68 ± 0.31^bcd^	7.60 ± 0.53^ cd^	2.93 ± 0.21^b^
* C. sinuosa* + 150 mM NaCl	6.47 ± 0.31^j^	3.53 ± 0.15^ef^	5.27 ± 0.45^a^	8.03 ± 0.48^c^	3.13 ± 0.25^b^
*Pandia pavonica*	11.23 ± 0.38^ab^	5.07 ± 0.57^a^	3.83 ± 0.35^efg^	5.83 ± 0.23^ g^	1.71 ± 0.12^f^
* P. pavonica* + 50 mM NaCl	8.97 ± 0.21^e^	3.93 ± 0.25^de^	3.87 ± 0.21^efg^	6.30 ± 0.46^ fg^	2.17 ± 0.12^ cd^
* P. pavonica* + 100 mM NaCl	7.30 ± 0.46^ g^	3.86 ± 0.22^de^	5.03 ± 0.47^abc^	7.07 ± 0.74^de^	2.88 ± 0.28^b^
* P. pavonica* + 150 mM NaCl	6.77 ± 0.25^j^	3.33 ± 0.40^ fg^	5.20 ± 0.36^ab^	7.61 ± 0.40^ cd^	3.09 ± 0.27^b^
Significance					
Salinity (S)	1494.81 ***	64.48 ***	47.79 ***	110.24 ***	113.17 ***
Seaweed extracts (SWEs)	9.67 ***	7.81 ***	4.06 *	48.66 ***	3.69 *
(S × SWEs)	23.79 ***	0.63 ns	3.12 **	5.22 ***	0.68 ns

Numbers represent the *F* value at 5% significance level. *ns* = non-significant

^*^P ˂ 0.05

^**^P ˂ 0.01

^***^P ˂ 0.001

The low molecular weight osmolytes proline and glycinebetaine (GB) were shown to be modulated by salinity and priming treatments in this study. Both molecules showed a progressive increase by increasing salinity level, as the salt concentration of 150 mM had resulted in 57.69 and 97.22% increases above the control level in proline and GB, respectively. All SWEs resulted in a decrease in proline levels as compared to control pea seedlings in the case of single priming treatments. The most marked decrease was perceived when the seedlings were pretreated with *P. pavonica* seaweed extract. Nonetheless, *S. vulgar* extract boosted GB levels marginally, whereas *C. sinousa* and *P. pavonica* extracts decreased it. Furthermore, increased accumulation of proline and GB owing to salt stress was demonstrated to be reduced by the combined treatments of salinity and seaweed extracts.

The two-way ANOVA of the physiological traits investigated in this study revealed that single treatments of salinity and SWEs significantly (*P* ˂ 0.05) affected all parameters, but the combined interaction of both treatments significantly affected Chl a, carotenoids, and proline levels, but non-significantly (*P* > 0.05) affected Chl b and GB levels.

### Protein SDS-PAGE variations

**Fig. 1:**
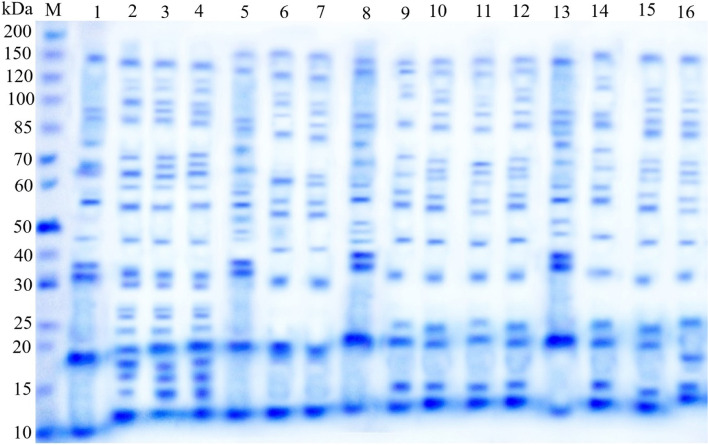
SDS-PAGE protein profile of 21-day-old pea seedlings treated with NaCl and SWEs (*S. vulgare*, *C. sinuosa*, and *P. pavonica*) single and combined treatments. kDa = kilo Dalton; M = marker protein; 1 = control; 2 = 50 mM NaCl; 3 = 100 mM NaCl; 4 = 150 mM NaCl; 5 = *S. vulgare* extract; 6 = *S. vulgare* extract + 50 mM NaCl; 7 = *S. vulgare* extract + 100 mM NaCl; 8 = *S. vulgare extract* + 150 mM NaCl; 9 = *C. sinuosa* extract; 10 = *C. sinuosa* extract + 50 mM NaCl; 11 = *C. sinuosa extract* + 100 mM NaCl; 12 = *C. sinuosa* extract + 150 mM NaCl; 13 = *P. pavonica* extract; 14 = *P. pavonica* extract + 50 mM NaCl; 15 = *P. pavonica* extract + 100 mM NaCl; 16 = *P. pavonica* extract + 150 mM NaCl

In Fig. 1 and Supplementary Table S1, the presence-absence score of protein SDS-PAGE profile of pea seedlings under NaCl salinity stress and priming with SWEs as well as their combinations is shown. Pea SDS–PAGE protein profiles revealed 32 protein bands with molecular weights ranging from 12 to 179 kDa. Eight bands with molecular weights of 143, 95, 65, 55, 46, 32, 20, and 12 kDa were monomorphic, and 21 bands were polymorphic (with a molecular weight range from 179 to 15 kDa). The number of protein bands expressed in the protein profile of control plants is only 11, which is much less than the number of protein bands expressed in the protein profiles of other treatments. The number of bands detected in the protein profile of seedlings exposed to NaCl treatments (19–21) was higher than the number of bands reported in the protein profile of seedlings primed with SWEs alone (15 bands) or in combination with NaCl treatments (12–17 bands). Six low-molecular-weight peptides with molecular weights of 60, 30, 27, 26, 18, and 16 kDa were produced as stress proteins in all NaCl-stressed seedlings but vanished in those primed with SWEs. In contrast, all salt-stressed seedlings, whether primed with SWEs or not, produced two peptides with molecular sizes of 119 and 110 kDa. Three peptides (72, 25, and 15 kDa) were eliminated by *S. vulgare* priming in all NaCl-stressed seedlings, three peptides (89, 77, and 35 kDa) were shared in the protein profiles of SWEs primed seedlings and control plants, and novel peptides of 58 and 47 kDa were expressed only by SWEs seed-priming. Nonetheless, 100 and 150 mM NaCl single treatments, as well as their combined interaction with SWEs priming elicited the expression of two distinct salt-responsive proteins of 100 and 70 kDa. Three bands were distinct to salt-stressed seedlings primed with SWEs, with molecular weights of 179 kDa for *S. vulgare*, 131 kDa for *C. sinuosa*, and 81 kDa for *P. pavonica*. In addition, priming with *S. vulgare*, *C. sinuosa*, and *P. pavonica* resulted in the expression of a specific distinct band of 51, 42, and 29 kDa, respectively (Supplementary Table S1).

### ISSR fingerprinting polymorphism

**Fig. 2:**
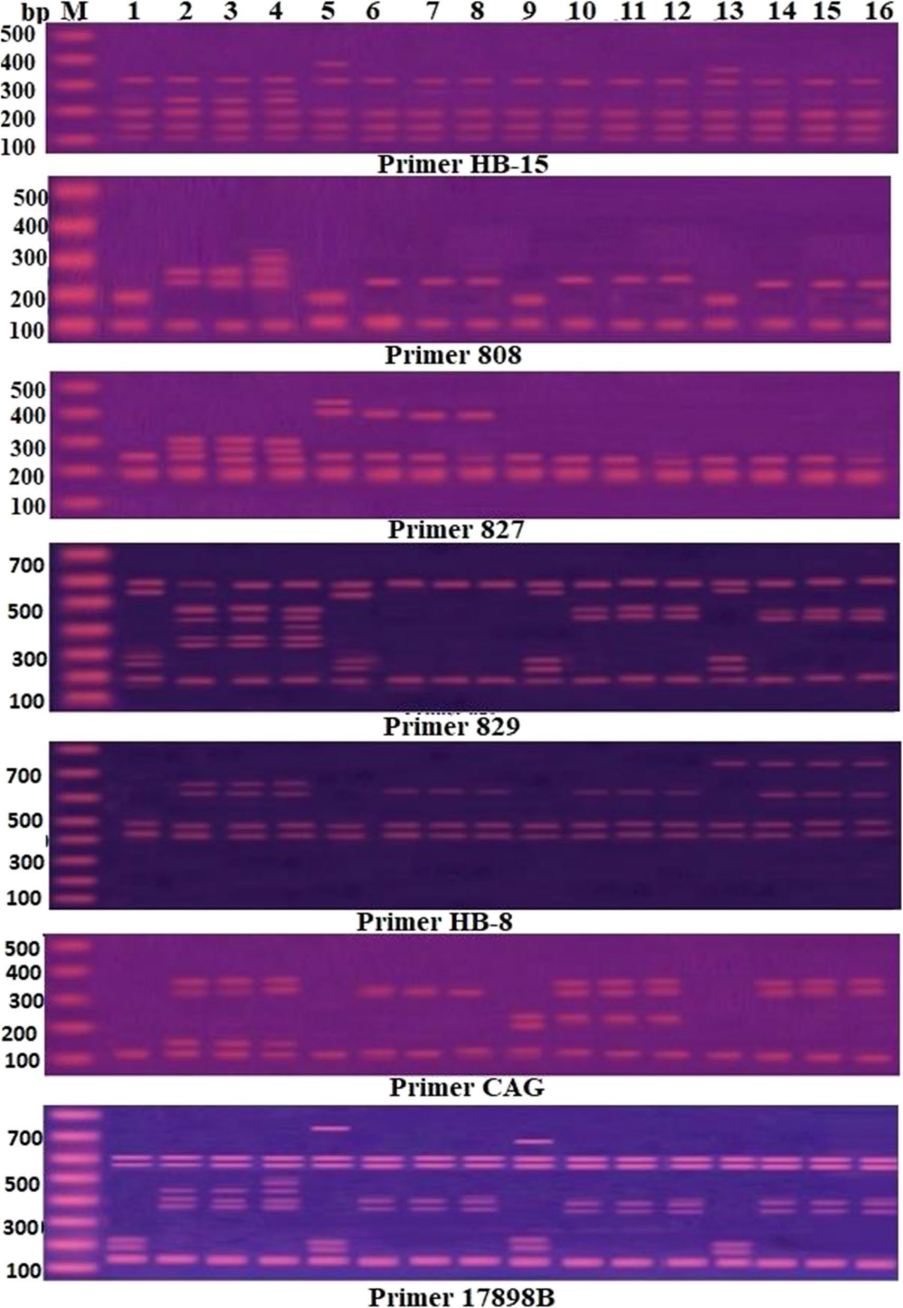
ISSR fingerprinting profile produced by seven primers in 21-day-old pea seedlings treated with NaCl and SWEs (*S. vulgare*, *C. sinuosa*, and *P. pavonica*) single and combined treatments. M = DNA molecular marker (bP); 1 = control; 2 = 50 mM NaCl; 3 = 100 mM NaCl; 4 = 150 mM NaCl; 5 = *S. vulgare* extract; 6 = *S. vulgare* extract + 50 mM NaCl; 7 = *S. vulgare* extract + 100 mM NaCl; 8 = *S. vulgare extract* + 150 mM NaCl; 9 = *C. sinuosa* extract; 10 = *C. sinuosa* extract + 50 mM NaCl; 11 = *C. sinuosa extract* + 100 mM NaCl; 12 = *C. sinuosa* extract + 150 mM NaCl; 13 = *P. pavonica* extract; 14 = *P. pavonica* extract + 50 mM NaCl; 15 = *P. pavonica* extract + 100 mM NaCl; 16 = *P. pavonica* extract + 150 mM NaCl

The ISSR fingerprinting profiles generated by seven primers in NaCl-treated and seaweed extracts primed pea seedlings, either single or combined treatments, are shown in Fig. 2. Table 1 shows the number of markers, their kind (polymorphic (P), monomorphic (M), or unique (U)), and the polymorphism percentage. Under the three levels of salinity, eight bands with molecular sizes of 261 bp (for primer 808), 310 and 275 bp (for primer 827), 438 and 410 bp (for primer 829), 652 bp (for primer HB-8), 144 bp (for primer CGA), and 436 bp (for primer 17898B) were documented, but they were not reported in the ISSR fingerprinting after SWEs priming. Six bands, on the other hand, were recorded after a combination of NaCl stress and SWEs priming (230 bp with primer 808, 616 bp with primer HB-8, 361 and321 bp with primer CGA, 403 and 368 bp with primer 17898B). Five bands were recorded in the control and after SWEs priming treatments, but were not observed in the ISSR fingerprinting of salt-stressed treatments (184 bp with primer 808, 533, and 473 bp with primer 829, 222, and 188 bp with primer 17898B). Finally, one band (405 bp with primer 827, 735 bp with primer HB-8, and 206 bp with primer CGA, respectively) was detected after *S. vulgare*, *P. pavonica*, and *C. sinuosa* priming single treatments (Fig. 2 and Supplementary Table S2).

Table 4 shows the distribution of ISSR markers by all primers in pea seedlings treated with four different NaCl-salinity levels (0, 50, 100, and 150 mM) or priming with SWEs (*S. vulgare*, *P. pavonica*, and *C. sinuosa*), either separately or in combination. The data revealed the presence of 25 polymorphic, 15 monomorphic, and 11 unique bands. The three salt stress treatments elicited a significantly larger number of ISSR bands than of control. In plants subjected to both 50 and 100 mM NaCl treatments, the number of ISSR bands rose from 20 in control plants to 32. However, 36 ISSR bands were induced in seedlings exposed to 150 mM NaCl, including four unique bands with sizes of 303 bp (primer 808), 265 bp (primer HB-15), 361 bp (primer 829), and 472 bp (primer 17898B). When compared to the control, priming with SWEs elicited more bands, although about ten bands induced by salinity treatments were not identified in priming and salt-stressed treatments. Priming with SWEs resulted in the development of seven distinct bands: four with *S. vulgare* extract (463 bp with primer 827, 384 bp with primer HB-15, 232 bp with primer 829, and 737 bp with primer 17898B), two with *C. sinuosa*, and one with *P. pavonica*. *S. vulgare* extract produced four unique bands, whereas *C. sinuosa* extract priming induced two unique bands (240 bp with primer CGA and 674 bp with primer 17898B), and *P. pavonica* extract induced one unique band (356 bp with primer HB-15).

**Table 4:** Number and types of ISSR bands reported in 21-day-old pea seedlings treated with NaCl and SWEs (*S. vulgare*, *C. sinuosa*, and *P. pavonica*) single and combined treatments

Band type and number	Treatments															
	Control	50 mM NaCl	100 mM NaCl	150 mM NaCl	*S. vulgare*	*S. v*. + 50 mM NaCl	*S. v.* + 10 mM NaCl	*S. v.* + 150 mM NaCl	*C. sinuosa*	C. s. + 50 mM NaCl	C. s. + 100 mM NaCl	C. s. + 150 mM NaCl	*P. pavonica*	*P. p.* + 50 mM NaCl	*P. p* + 100 mM NaCl	*P. p*. + 150 mM NaCl
Number of bands by all primers																
Total markers	20	32	32	36	25	22	22	22	23	24	24	24	22	24	24	24
Unique	–	–	–	4	4	–	–	–	2	–	–	–	1	–	–	–
Monomorphic	15	15	15	15	15	15	15	15	15	15	15	15	15	15	15	15
Polymorphic	5	17	17	15	6	7	7	7	6	9	9	9	6	9	9	9

**Fig. 3:**
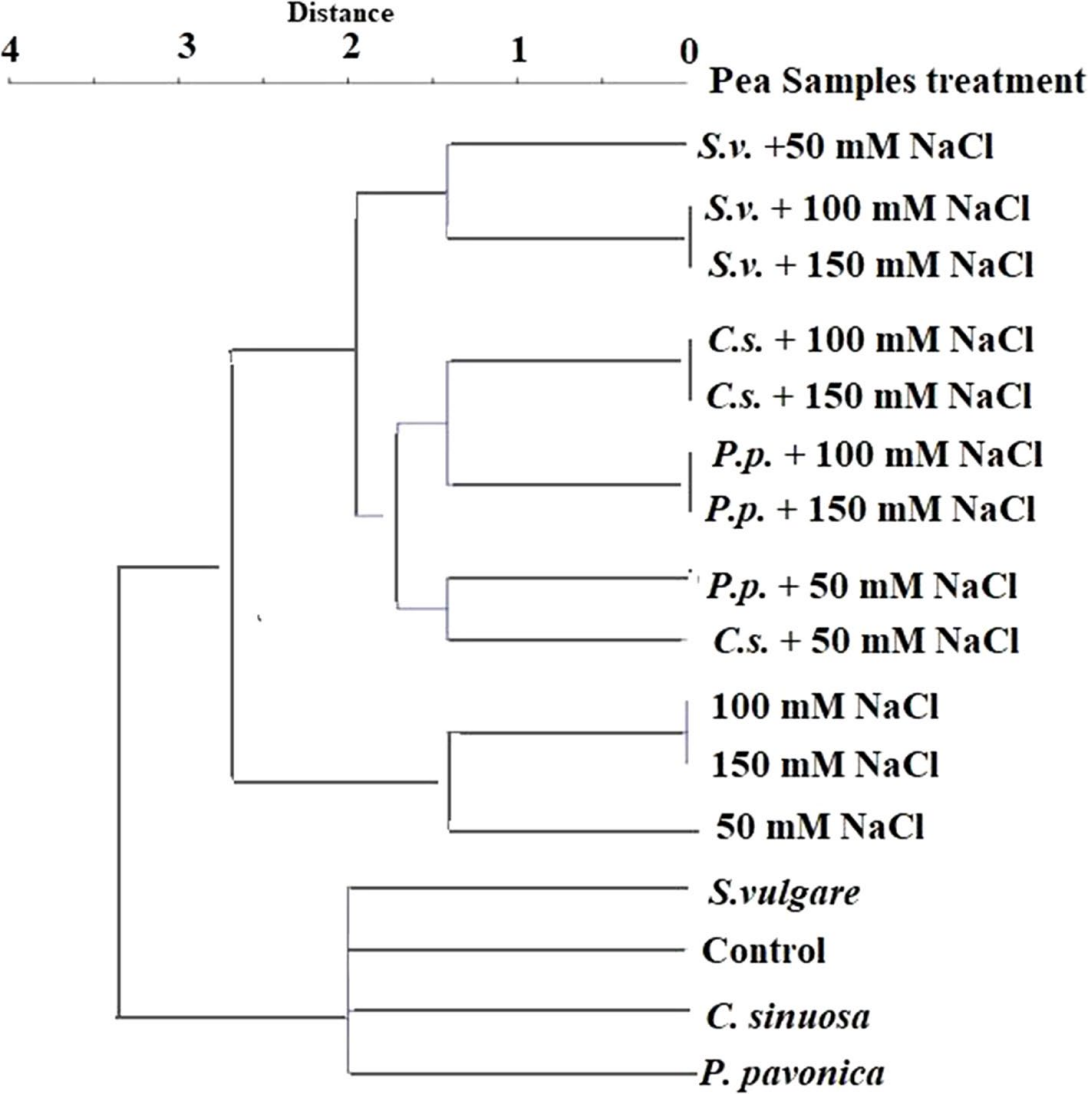
Cluster tree depicting the differentiation of pea seedlings treated with NaCl (0, 50, 100, and 150 mM), priming with SWEs (*S. vulgare*, *C. sinuosa*, and *P. pavonica*) as single or combined treatments based on the variation in SDS-PAGE protein profile and ISSR DNA fingerprinting

The dendrogram shown in Fig. 3 represents the cluster analysis of protein and ISSR data differentiation of the control seedlings and the 15 seedling samples representing NaCl-salinity treatments, SWEs priming, and their combinations. The control plants and plants developed from SWEs-primed seeds were grouped together, whereas the plants subjected to the NaCl-salt treatments were categorized into another group. Plants primed with *S. vulgare* and subjected to 50, 100, and 150 mM NaCl treatments formed a distinct group, whereas those primed with *P. pavonica* and *C. sinuosa* and exposed to 50 mM NaCl were distinguished from those primed with these two seaweed extracts and subjected to 100 and 150 mM NaCl. The full similarity of plants subjected to 100 and 150 mM NaCl, with or without seed priming with SWEs, is a significant observation (Fig. 3).

## Discussion

The current study reported that NaCl stress affected pea seed germination by delaying seedling emergence, which resulted in a lower germination percentage. Katembe et al. [49] attributed the inhibition of seed germination to the low osmotic potential created around the seed by the salt solution. It has also been linked to the toxicity of NaCl ions, which hinder cells from absorbing water [6]. The physiological state of the seed at the early stages of germination, which contributes to the seed and seedling performance, is determined, at least in part, by the stored mRNAs translated to produce protein enzymes upon seed imbibition for germination. Massive transcriptome alterations occur shortly after water consumption, influenced by ambient temperature, light conditions, and plant hormones, including abscisic acid and gibberellins [50, 51]. According to Liu et al. [52], NaCl-induced germination suppression was caused by the downregulation of gibberellin genes, which resulted in a lack of gibberellic acid and a reduction in α-amylase activity.

Because roots absorb water through direct contact with the soil, and shoots facilitate water supply throughout the plant, shoot and root length are critical characteristics of salt tolerance [53]. At high salt levels (150 mM NaCl), NaCl treatments resulted in a considerable reduction in pea seedling length in the current study. According to Naseer et al. [54], seedling length reduction is caused by an excessive accumulation of salt in the cell wall, which reduces cell wall flexibility and alters metabolic activities. Salt stress, according to Naz et al. [55], reduced the effectiveness of translocation and assimilation of stored nutrients, resulting in a decrease in pea seedling growth. In comparison to the control, the current findings demonstrated that the fresh weight of pea seedlings decreased as salt stress levels increased. This could be related to the stress that salt has on seedling metabolic activities, which diminish water potential due to ionic and osmotic stress [6]. 

As a result of the production of reactive oxygen species (ROS), plants are vulnerable to oxidative stress. Several mechanisms have been proposed to explain how plants generate a variety of physiological and biochemical adaptations to empower them to survive properly in salty conditions. Exogenous protectants, such as osmoregulators [56], plant hormones [57], antioxidants [58], and seaweed extracts [24] for example, were shown to be effective in reducing the deleterious effects of salinity on plant growth. The deleterious effects of NaCl have also been attributed to changes in osmotic potential caused by decreased water availability [6]. The use of seaweed priming for environmental stress tolerance has been gaining traction in agricultural production owing to its unique bioactive components such as essential macro and micronutrients, growth regulators, choline chloride, and glycine betaine [17, 59]. These seaweeds have plant growth stimulatory effects as well as elicitor activity, which causes plants to mount defense mechanisms in response to pests, diseases, and abiotic challenges such as drought and salinity [18]. When pea seeds were exposed to NaCl salinity, priming with seaweed extracts (SWEs) resulted in a significant increase in germination rate and seedling fresh weight when compared to their corresponding controls. This is because minerals, ascorbic acid, proline, and glutathione, among other biological constituents in seaweed extract, may be involved in the induction of antioxidant enzymes that diminish ROS levels in salinity-stressed plants [26, 60]. 

Extreme environmental factors such as salinity, water stress, temperature, and heavy metals are extremely stressful to photosynthetic machinery. Increased chlorophyllase activity, which deteriorates pigment proteins and eventually reduces chlorophyll content in plants, could explain the salt-induced reduction in chlorophyll content described in this work. Salinity-induced chlorophyll reduction could be largely related to either the slowdown in chlorophyll biosynthesis or accelerated enzymatic chlorophyll degradation [61], as well as the degeneration of thylakoid membrane and the breakdown of chlorophyll via oxidative damage-induced ROS, in addition to the modifications in chlorophyll protein complexes [62]. Furthermore, salinity has been shown to reduce the expression of several Mg-chelatase subunit-encoding genes and the concentration of chlorophyll biosynthetic intermediates [63].

Carotenoids protect plant cells and organelles from ROS by quenching singlet oxygen molecules and scavenging free radicals [64]. The results of [65] working on guava and Ben-Abdallah et al. [66] working on nightshade support our findings of elevated concentrations of carotenoids in pea grown in saline conditions. The carotenoids’ biosynthetic pathway, which is mediated by abscisic acid, has been reported to be triggered as a defensive response against ROS produced at salt-stress conditions [67]. According to Li et al. [68], the overexpression of genes involved in enhanced carotenoids accumulation could allow plants to develop higher efficiency to use enzymes that eliminate ROS under salt-stress conditions.

The results of this study also showed that the osmoregulatory molecules proline and glycinebetaine (GB) were accumulated in pea seedlings in response to salinity stress. Proline is a versatile amino acid that increases under salt stress. It is an essential osmoprotectant that defends plants from oxidative damage by preserving proteins and cell membranes, neutralizing free radicals, establishing cellular homeostasis, and sustaining redox potential [69]. As the protein synthesis machinery is shifted to proline accumulation, the faster rate of protein hydrolysis and the slower rate of proline degradation could explain the increased proline concentration caused by salinity [70]. Under salt stress, GB was reported to play many roles as a compatible solute in osmotic adjustment, safeguarding both protein and membrane functions from adverse levels of Na and Cl, stabilizing enzyme and protein structures, and removal of excess ROS [71]. Furthermore, GB could indirectly trigger changes in coenzyme turnover, which is critical for maintaining photosynthesis and respiration rates under stressful conditions [72].

Priming pea seeds with liquid SWEs positively affected the concentration of the photosynthetic pigments in the present study. The plant growth-promoting compounds and high mineral composition of these seaweed extracts may account for the stimulatory effects of SWEs priming on chlorophyll and carotenoids concentrations in pea leaves [73]. In addition, during salt stress, SWEs-treated pea seedlings had increased levels of photosynthetic pigments. This suggests that SWEs conferred salt stress endurance in pea plants and that the bioactive compounds delivered from these SWEs may offer additional salinity tolerance and improved plant performance [24]. Furthermore, bioactive compounds contained in SWEs, including amino acids, betaines, and minerals, could account for their role in reducing pigment breakdown and increasing photosynthetic potential in stressed plants [74].

The reduction in proline and GB concentrations caused by SWE priming could be linked to improved nitrogen metabolism, implying that nitrogenous substances were integrated into plant development pathways [75]. These findings suggest that SWE-primed plants were less stressed than control and salt-stressed plants, resulting in sustained growth. The decline in proline and GB following SWEs priming in salts-stressed plants reported in this study is in agreement with the findings of Kasim et al. [24]. They ascribed the decrement in proline concentration to the increase in protein, which could imply that proline was integrated into protein synthesis. However, the lower concentration of GB in this study could be explained by the fact that SWEs are rich in GB, which minimizes the demands of plants to consume the internal plant energy in endogenous GB biosynthesis.

Total protein synthesis in plant cells typically decreases when exposed to NaCl salinity; however, exposure to salt stress enhanced the accumulation of salt-specific proteins in the current study, as evidenced by changes in SDS-PAGE profiles. Similarly, Win and Oo [31] found that the salt-stress-induced alterations in protein profiles vary according to the salinity tolerance of *Vigna mungo* varieties. Some proteins are expressed in salt-stressed plants, likely as a result of transcriptional upregulation contributing to the synthesis of new stress proteins [76]. Furthermore, in plants exposed to salinity, protein accumulation may act as an energy store, permitting them to manipulate their osmotic potential [31].

In the current study, NaCl treatments resulted in the accumulation of two new proteins with molecular sizes of 70 and 100 kDa, and priming of pea seeds with *S. vulgare*, *C. sinuosa*, and *P. pavonica* SWEs resulted in the production of three new proteins with molecular sizes of 51, 42, and 29 KDa, respectively. The newly synthesized polypeptides in the stressed seedlings could demonstrate that the cell is protecting itself against the salt's harmful effects [77]. One or more bioactive compounds in seaweed extracts, in addition to other growth-promoting substances, may cause upregulation of polypeptide synthesis by activating protein biosynthesis enzymes as a defense mechanism against stress [18]. Additionally, the presence of novel polypeptides could be linked to NaCl ion toxicity, which can trigger signaling processes that affect gene expression [78]. Salinity can also cause chromosomal rearrangement, strand breaks, base deletions, pyrimidine dimers, mutations, cross-links, and base alterations in DNA, resulting in genotoxic impairment and structural modifications [79]. However, pea seeds priming with SWEs caused the elimination of some polypeptides, which could be the result of a cascade of biochemical and molecular changes in primed-stressed plants attempting to adjust to the change in gene expression [80]. 

The number of ISSR markers increased significantly in the 150 mM NaCl-stressed seedlings, as it increased from 20 in the control seedlings to 36 in NaCl-stressed seedlings. In addition, 150 mM NaCl treatment provoked the development of four unique bands. Nonetheless, priming pea seeds with SWEs caused more bands than the control treatment; however, many salinity-induced bands disappeared when SWEs priming was used alone or in combination with NaCl treatments, even though seven unique bands were induced by priming with SWEs. Our findings demonstrated that NaCl-stress and SWEs (*S. vulgare*, *C. sinuosa*, and *P. pavonica*) priming, in single or combination treatments, resulted in stimulation of genetic divergence in pea seedlings as revealed by the seven primers used. In this respect, ISSR fingerprints were detected to be cultivar-specific markers in a variety of wheat and barley genotypes under salt stress, which can be used to identify stress resistance genotypes as well as plant germplasm management and conservation. The unique bands created by the primers served as a unique identifier for salinity tolerance [39, 40].

The variability in ISSR fingerprinting patterns; the presence or absence of DNA fragments, could suggest DNA damage caused by point mutations as a result of ROS generation by salt stress [81]. Increased ROS may exacerbate genomic DNA damage, resulting in ISSR polymorphism. DNA is a common target for ROS, and it can be damaged to create a variety of genotoxic endpoints. ROS assaults DNA and causes alterations to DNA bases, such as single-strand and double-strand breaks in the DNA molecule that are not always repairable [82]. Under ROS stress, DNA double-strand breaks are one of the most common types of DNA damage, resulting in genome instability and causing DNA fragmentation [83]. The ISSR markers fingerprinting variation showed an increase in polymorphism as the concentration of NaCl increased in the current study, and thus could be used to measure the qualitative genotoxic activity of salinity and other environmental stress factors such as drought, heat, pollutants, and so on, to identify target genes for specific genotoxic agents.

## Conclusion

Seaweed priming of pea seeds increased germination, seedling length, and fresh weight while also promoting the biosynthesis of photosynthetic pigments, new peptides, and ISSR markers. Proteins produced by priming seeds with SWEs could help plants adapt to the imposed salinity stress. The variability of the ISSR markers indicates DNA damage that could be attributable to point mutations as a result of DNA damage caused by ROS produced by salt stress. Also, when exposed to salt stress, the seaweed extract of *S. vulgare* was the most efficient in promoting pea seed germination and seedling growth. SWEs have the potential to be good suppliers of highly bioactive compounds, offering them a promising approach for alleviating salt stress. More research is needed on the role of SWEs to better understand their potential for relieving salt stress and to comprehend more about the physiological and molecular mechanisms that reflect their involvement in plant salt tolerance.

## Supplementary Information

**Additional file 1:** **Table S1.** The presence-absence score of leaf proteins SDS-PAGE profile of pea seedlings under salinity stress treatments and priming with extract of the seaweeds *Sargassum vulgare* (S) *Colpomenia* *sinuosa *(C) and *Pandia pavonica* (P), and their combinations. Band type*: Polymorphic (P), Monomorphic (M) and Unique (U). **Table S2.** ISSR fingerprinting score produced by the DNA of 21-days-old pea leaves using the seven primers given in Table 1, grown under 50 ,100, and 150 mM NaCl in combination with marine brown seaweeds (*Sargassum vulgare* (S), *Colpomenia sinuosa* (C), and *Pandia pavonica* (P)).

## Data Availability

All data supporting the conclusions of this article are provided with the article and its supplementary information files.

## Abbreviations

SWEs: Seaweed extracts
ISSR: Inter-simple sequence repeats
mM: Millimolar
SDS-PAGE: Sodium dodecyl sulfate–polyacrylamide gel electrophoresis
w/w: Weight per weight
EC: Electrical conductivity
dS.m^−1^: DeciSiemens per meter
GB: Glycinebetaine
DM: Dry matter
kDa: Kilodalton
TAE buffer: Tris–acetate-EDTA buffer
bp: Base pair
