# Supplementary material for: Growth, physiological, and molecular responses of three phaeophyte extracts on salt-stressed pea (Pisum sativum L.) seedlings
Source: J Genet Eng Biotechnol. 2023 Mar 16;21:32. doi: 10.1186/s43141-023-00483-z (PMC10020410; doi:10.1186/s43141-023-00483-z)
Supplement: Supplementary file 1 — Additional file 1: Table S1. The presence-absence score of leaf proteins SDS-PAGE profile of pea seedlings under salinity stress treatments and priming with extract of the seaweeds Sargassum vulgare (S) Colpomenia sinuosa (C) and Pandia pavonica (P), and their combinations. Band type*: Polymorphic (P), Monomorphic (M) and Unique (U). Table S2. ISSR fingerprinting score produced by the DNA of 21-days-old pea leaves using the seven primers given in Table 1, grown under 50 ,100, and 150 mM NaCl in combination with marine brown seaweeds (Sargassum vulgare (S), Colpomenia sinuosa (C), and Pandia pavonica (P)). [file 43141_2023_483_MOESM1_ESM.docx]

**Table S1.** The presence absence score of leaf protein SDS-PAGE profile of pea seedlings under salinity stress treatments and priming with extract of the seaweeds *Sargassum vulgare* (S) *Colpomenia* *sinuosa* (C) and *Pandia pavonica* (P) and their combinations. Band type*: Polymorphic (P), Monomorphic(M) and Unique (U).

| Band number | Molecular weight (kDa) | NaCl and seaweeds extract priming treatments | | | | | | | | | | | | | | | | Band type * |
| --- | --- | --- | --- | --- | --- | --- | --- | --- | --- | --- | --- | --- | --- | --- | --- | --- | --- | --- |
|  |  | Control | 50 mM NaCl | 100 mM NaCl | 150 mM NaCl | *S. vulgare* | *S. v.* + 50 mM NaCl | *S. v*. + 100 mM NaC | *S. v.* +150 mM NaCl | *C. sinuosa* | *C. s*. +50 m M NaC | *C. s*. +100 mM NaCl | *C. s*.+150 mM NaCl | *P. pavonica* | *P. p*. + 50 mM NaCl | *P. p.* +100 mM NaCl | *P. p.* +150 mM NaCl |  |
| 01 | 179 | 0 | 0 | 0 | 0 | 1 | 1 | 1 | 1 | 0 | 0 | 0 | 0 | 0 | 0 | 0 | 0 | P |
| 02 | 143 | 1 | 1 | 1 | 1 | 1 | 1 | 1 | 1 | 1 | 1 | 1 | 1 | 1 | 1 | 1 | 1 | M |
| 03 | 131 | 0 | 0 | 0 | 0 | 0 | 0 | 0 | 0 | 1 | 1 | 1 | 1 | 0 | 0 | 0 | 0 | P |
| 04 | 119 | 0 | 1 | 1 | 1 | 0 | 1 | 1 | 1 | 0 | 1 | 1 | 1 | 0 | 1 | 1 | 1 | P |
| 05 | 110 | 0 | 1 | 1 | 1 | 0 | 1 | 1 | 1 | 0 | 1 | 1 | 1 | 0 | 1 | 1 | 1 | P |
| 06 | 100 | 0 | 0 | 1 | 1 | 0 | 0 | 1 | 1 | 0 | 0 | 1 | 1 | 0 | 0 | 1 | 1 | P |
| 07 | 95 | 1 | 1 | 1 | 1 | 1 | 1 | 1 | 1 | 1 | 1 | 1 | 1 | 1 | 1 | 1 | 1 | M |
| 08 | 89 | 1 | 0 | 0 | 0 | 1 | 0 | 0 | 0 | 1 | 0 | 0 | 0 | 1 | 0 | 0 | 0 | P |
| 09 | 81 | 0 | 0 | 0 | 0 | 0 | 0 | 0 | 0 | 0 | 0 | 0 | 0 | 1 | 1 | 1 | 1 | P |
| 10 | 77 | 1 | 0 | 0 | 0 | 1 | 0 | 0 | 0 | 1 | 0 | 0 | 0 | 1 | 0 | 0 | 0 | P |
| 11 | 72 | 0 | 1 | 1 | 1 | 0 | 0 | 0 | 0 | 0 | 1 | 1 | 1 | 0 | 1 | 1 | 1 | P |
| 12 | 70 | 0 | 0 | 1 | 1 | 0 | 0 | 1 | 1 | 0 | 0 | 1 | 1 | 0 | 0 | 1 | 1 | P |
| 13 | 65 | 1 | 1 | 1 | 1 | 1 | 1 | 1 | 1 | 1 | 1 | 1 | 1 | 1 | 1 | 1 | 1 | M |
| 14 | 60 | 0 | 1 | 1 | 1 | 0 | 0 | 0 | 0 | 0 | 0 | 0 | 0 | 0 | 0 | 0 | 0 | P |
| 15 | 58 | 0 | 0 | 0 | 0 | 1 | 1 | 1 | 1 | 1 | 1 | 1 | 1 | 1 | 1 | 1 | 1 | P |
| 16 | 55 | 1 | 1 | 1 | 1 | 1 | 1 | 1 | 1 | 1 | 1 | 1 | 1 | 1 | 1 | 1 | 1 | M |
| 17 | 51 | 0 | 0 | 0 | 0 | 1 | 0 | 0 | 0 | 0 | 0 | 0 | 0 | 0 | 0 | 0 | 0 | U |
| 18 | 47 | 0 | 0 | 0 | 0 | 1 | 0 | 0 | 0 | 1 | 0 | 0 | 0 | 1 | 0 | 0 | 0 | P |
| 19 | 46 | 1 | 1 | 1 | 1 | 1 | 1 | 1 | 1 | 1 | 1 | 1 | 1 | 1 | 1 | 1 | 1 | M |
| 20 | 42 | 0 | 0 | 0 | 0 | 0 | 0 | 0 | 0 | 1 | 0 | 0 | 0 | 0 | 0 | 0 | 0 | U |
| 21 | 35 | 1 | 0 | 0 | 0 | 1 | 0 | 0 | 0 | 1 | 0 | 0 | 0 | 1 | 0 | 0 | 0 | P |
| 22 | 32 | 1 | 1 | 1 | 1 | 1 | 1 | 1 | 1 | 1 | 1 | 1 | 1 | 1 | 1 | 1 | 1 | M |
| 23 | 30 | 0 | 1 | 1 | 1 | 0 | 0 | 0 | 0 | 0 | 0 | 0 | 0 | 0 | 0 | 0 | 0 | P |
| 24 | 29 | 0 | 0 | 0 | 0 | 0 | 0 | 0 | 0 | 0 | 0 | 0 | 0 | 1 | 0 | 0 | 0 | U |
| 25 | 27 | 0 | 1 | 1 | 1 | 0 | 0 | 0 | 0 | 0 | 0 | 0 | 0 | 0 | 0 | 0 | 0 | P |
| 26 | 26 | 0 | 1 | 1 | 1 | 0 | 0 | 0 | 0 | 0 | 0 | 0 | 0 | 0 | 0 | 0 | 0 | P |
| 27 | 25 | 0 | 1 | 1 | 1 | 0 | 0 | 0 | 0 | 0 | 1 | 1 | 1 | 0 | 1 | 1 | 1 | P |
| 28 | 20 | 1 | 1 | 1 | 1 | 1 | 1 | 1 | 1 | 1 | 1 | 1 | 1 | 1 | 1 | 1 | 1 | M |
| 29 | 18 | 0 | 1 | 1 | 1 | 0 | 0 | 0 | 0 | 0 | 0 | 0 | 0 | 0 | 0 | 0 | 0 | P |
| 30 | 16 | 0 | 1 | 1 | 1 | 0 | 0 | 0 | 0 | 0 | 0 | 0 | 0 | 0 | 0 | 0 | 0 | P |
| 31 | 15 | 0 | 1 | 1 | 1 | 0 | 0 | 0 | 0 | 0 | 1 | 1 | 1 | 0 | 1 | 1 | 1 | P |
| 32 | 12 | 1 | 1 | 1 | 1 | 1 | 1 | 1 | 1 | 1 | 1 | 1 | 1 | 1 | 1 | 1 | 1 | M |
|  | | 11 | 19 | 21 | 21 | 15 | 12 | 14 | 14 | 15 | 15 | 17 | 17 | 15 | 15 | 17 | 17 |  |

**Table S2.** ISSR fingerprinting score produced by the DNA of 21-days-old pea leaves using the seven primers given in Table 1, grown under 50 ,100,150 mM NaCl exposure in combination with marine brown seaweeds (*Sargassum vulgare* (S) *Colpomenia sinuosa* (C) and *Pandia pavonica* (P)

| Band number | Molecular size (bp) | **Treatments** | | | | | | | | | | | | | | | | Band type***** |
| --- | --- | --- | --- | --- | --- | --- | --- | --- | --- | --- | --- | --- | --- | --- | --- | --- | --- | --- |
|  |  | Control | 50 mM NaCl | 100 mM NaCl | 150 mM NaCl | *S. vulgare* | *S. v*. + 50 mM NaCl | *S. v.* +100 mM NaCl | *S. v.* +150 mM NaCl | *C. sinuosa* | *C. s*.+50 m M NaCl | *C. s*.+100 mM NaCl | *C. s*.+150 mM NaCl | *P. pavonica* | *P. p.* + 50 mM NaCl | *P. p* +100 mM NaCl | *P. p*.+150 mM NaCl |  |
| **1-Primer 808** | | | | | | | | | | | | | | | | | | |
| **1** | **303** | 0 | 0 | 0 | 1 | 0 | 0 | 0 | 0 | 0 | 0 | 0 | 0 | 0 | 0 | 0 | 0 | U |
| **2** | **261** | 0 | 1 | 1 | 1 | 0 | 0 | 0 | 0 | 0 | 0 | 0 | 0 | 0 | 0 | 0 | 0 | P |
| **3** | **230** | 0 | 1 | 1 | 1 | 0 | 1 | 1 | 1 | 0 | 1 | 1 | 1 | 0 | 1 | 1 | 1 | P |
| **4** | **184** | 1 | 0 | 0 | 0 | 1 | 0 | 0 | 0 | 1 | 0 | 0 | 0 | 1 | 0 | 0 | 0 | P |
| **5** | **96** | 1 | 1 | 1 | 1 | 1 | 1 | 1 | 1 | 1 | 1 | 1 | 1 | 1 | 1 | 1 | 1 | M |
| **2- primer 827** | | | | | | | | | | | | | | | | | | |
| **6** | **463** | 0 | 0 | 0 | 0 | 1 | 0 | 0 | 0 | 0 | 0 | 0 | 0 | 0 | 0 | 0 | 0 | U |
| **7** | **405** | 0 | 0 | 0 | 0 | 1 | 1 | 1 | 1 | 0 | 0 | 0 | 0 | 0 | 0 | 0 | 0 | P |
| **8** | **310** | 0 | 1 | 1 | 1 | 0 | 0 | 0 | 0 | 0 | 0 | 0 | 0 | 0 | 0 | 0 | 0 | P |
| **9** | **275** | 0 | 1 | 1 | 1 | 0 | 0 | 0 | 0 | 0 | 0 | 0 | 0 | 0 | 0 | 0 | 0 | P |
| **10** | **243** | 1 | 1 | 1 | 1 | 1 | 1 | 1 | 1 | 1 | 1 | 1 | 1 | 1 | 1 | 1 | 1 | M |
| **11** | **191** | 1 | 1 | 1 | 1 | 1 | 1 | 1 | 1 | 1 | 1 | 1 | 1 | 1 | 1 | 1 | 1 | M |
| **3- Primer HB-15** | | | | | | | | | | | | | | | | | | |
| **12** | **384** | 0 | 0 | 0 | 0 | 1 | 0 | 0 | 0 | 0 | 0 | 0 | 0 | 0 | 0 | 0 | 0 | U |
| **13** | **356** | 0 | 0 | 0 | 0 | 0 | 0 | 0 | 0 | 0 | 0 | 0 | 0 | 1 | 0 | 0 | 0 | U |
| **14** | **316** | 1 | 1 | 1 | 1 | 1 | 1 | 1 | 1 | 1 | 1 | 1 | 1 | 1 | 1 | 1 | 1 | M |
| **15** | **265** | 0 | 0 | 0 | 1 | 0 | 0 | 0 | 0 | 0 | 0 | 0 | 0 | 0 | 0 | 0 | 0 | U |
| **16** | **235** | 0 | 1 | 1 | 1 | 0 | 0 | 0 | 0 | 0 | 0 | 0 | 0 | 0 | 0 | 0 | 0 | P |
| **17** | **194** | 1 | 1 | 1 | 1 | 1 | 1 | 1 | 1 | 1 | 1 | 1 | 1 | 1 | 1 | 1 | 1 | M |
| **18** | **138** | 1 | 1 | 1 | 1 | 1 | 1 | 1 | 1 | 1 | 1 | 1 | 1 | 1 | 1 | 1 | 1 | M |
| **19** | **103** | 1 | 1 | 1 | 1 | 1 | 1 | 1 | 1 | 1 | 1 | 1 | 1 | 1 | 1 | 1 | 1 | M |
| **4- Primer 829** | | | | | | | | | | | | | | | | | | |
| **20** | **582** | 1 | 1 | 1 | 1 | 1 | 1 | 1 | 1 | 1 | 1 | 1 | 1 | 1 | 1 | 1 | 1 | M |
| **21** | **533** | 1 | 0 | 0 | 0 | 1 | 0 | 0 | 0 | 1 | 0 | 0 | 0 | 1 | 0 | 0 | 0 | P |
| **22** | **473** | 1 | 0 | 0 | 0 | 1 | 0 | 0 | 0 | 1 | 0 | 0 | 0 | 1 | 0 | 0 | 0 | P |
| **23** | **438** | 0 | 1 | 1 | 1 | 0 | 0 | 0 | 0 | 0 | 0 | 0 | 0 | 0 | 0 | 0 | 0 | P |
| **24** | **410** | 0 | 1 | 1 | 1 | 0 | 0 | 0 | 0 | 0 | 0 | 0 | 0 | 0 | 0 | 0 | 0 | P |
| **25** | **361** | 0 | 0 | 0 | 1 | 0 | 0 | 0 | 0 | 0 | 0 | 0 | 0 | 0 | 0 | 0 | 0 | U |
| **26** | **329** | 0 | 1 | 1 | 1 | 0 | 0 | 0 | 0 | 0 | 1 | 1 | 1 | 0 | 1 | 1 | 1 | P |
| 27 | **262** | 0 | 1 | 1 | 1 | 0 | 0 | 0 | 0 | 0 | 1 | 1 | 1 | 0 | 1 | 1 | 1 | P |
| **28** | **232** | 0 | 0 | 0 | 0 | 1 | 0 | 0 | 0 | 0 | 0 | 0 | 0 | 0 | 0 | 0 | 0 | U |
| **29** | **178** | 1 | 1 | 1 | 1 | 1 | 1 | 1 | 1 | 1 | 1 | 1 | 1 | 1 | 1 | 1 | 1 | M |
| **5- primer HB-8** | | | | | | | | | | | | | | | | | | |
|  |  | **0** | **50** | **100** | **150** | **Sv** | **50** | **100** | **150** | **Cv** | **50** | **100** | **150** | **pp** | **50** | **100** | **150** |  |
| **30** | **735** | 0 | 0 | 0 | 0 | 0 | 0 | 0 | 0 | 0 | 0 | 0 | 0 | 1 | 1 | 1 | 1 | P |
| **31** | **652** | 0 | 1 | 1 | 1 | 0 | 0 | 0 | 0 | 0 | 0 | 0 | 0 | 0 | 0 | 0 | 0 | P |
| **32** | **616** | 0 | 1 | 1 | 1 | 0 | 1 | 1 | 1 | 0 | 1 | 1 | 1 | 0 | 1 | 1 | 1 | P |
| **33** | **466** | 1 | 1 | 1 | 1 | 1 | 1 | 1 | 1 | 1 | 1 | 1 | 1 | 1 | 1 | 1 | 1 | M |
| **34** | **413** | 1 | 1 | 1 | 1 | 1 | 1 | 1 | 1 | 1 | 1 | 1 | 1 | 1 | 1 | 1 | 1 | M |
| **6- primer CGA** | | | | | | | | | | | | | | | | | | |
| **35** | **361** | 0 | 1 | 1 | 1 | 0 | 1 | 1 | 1 | 0 | 1 | 1 | 1 | 0 | 1 | 1 | 1 | P |
| **36** | **321** | 0 | 1 | 1 | 1 | 0 | 1 | 1 | 1 | 0 | 1 | 1 | 1 | 0 | 1 | 1 | 1 | P |
| **37** | **240** | 0 | 0 | 0 | 0 | 0 | 0 | 0 | 0 | 1 | 0 | 0 | 0 | 0 | 0 | 0 | 0 | U |
| **38** | **206** | 0 | 0 | 0 | 0 | 0 | 0 | 0 | 0 | 1 | 1 | 1 | 1 | 0 | 0 | 0 | 0 | P |
| **39** | **144** | 0 | 1 | 1 | 1 | 0 | 0 | 0 | 0 | 0 | 0 | 0 | 0 | 0 | 0 | 0 | 0 | P |
| **40** | **114** | 1 | 1 | 1 | 1 | 1 | 1 | 1 | 1 | 1 | 1 | 1 | 1 | 1 | 1 | 1 | 1 | M |
| **7- Primer 17898B** | | | | | | | | | | | | | | | | | | |
| **41** | **737** | 0 | 0 | 0 | 0 | 1 | 0 | 0 | 0 | 0 | 0 | 0 | 0 | 0 | 0 | 0 | 0 | U |
| **42** | **674** | 0 | 0 | 0 | 0 | 0 | 0 | 0 | 0 | 1 | 0 | 0 | 0 | 0 | 0 | 0 | 0 | U |
| **43** | **602** | 1 | 1 | 1 | 1 | 1 | 1 | 1 | 1 | 1 | 1 | 1 | 1 | 1 | 1 | 1 | 1 | M |
| **44** | **565** | 1 | 1 | 1 | 1 | 1 | 1 | 1 | 1 | 1 | 1 | 1 | 1 | 1 | 1 | 1 | 1 | M |
| **45** | **472** | 0 | 0 | 0 | 1 | 0 | 0 | 0 | 0 | 0 | 0 | 0 | 0 | 0 | 0 | 0 | 0 | U |
| **46** | **436** | 0 | 1 | 1 | 1 | 0 | 0 | 0 | 0 | 0 | 0 | 0 | 0 | 0 | 0 | 0 | 0 | P |
| **47** | **403** | 0 | 1 | 1 | 1 | 0 | 1 | 1 | 1 | 0 | 1 | 1 | 1 | 0 | 1 | 1 | 1 | P |
| **48** | **368** | 0 | 1 | 1 | 1 | 0 | 1 | 1 | 1 | 0 | 1 | 1 | 1 | 0 | 1 | 1 | 1 | P |
| **49** | **222** | 1 | 0 | 0 | 0 | 1 | 0 | 0 | 0 | 1 | 0 | 0 | 0 | 1 | 0 | 0 | 0 | P |
| **50** | **188** | 1 | 0 | 0 | 0 | 1 | 0 | 0 | 0 | 1 | 0 | 0 | 0 | 1 | 0 | 0 | 0 | P |
| **51** | **135** | 1 | 1 | 1 | 1 | 1 | 1 | 1 | 1 | 1 | 1 | 1 | 1 | 1 | 1 | 1 | 1 | M |
|  |  |  |  |  |  |  |  |  |  |  |  |  |  |  |  |  |  |  |
| **Total** | | 20 | 32 | 32 | 36 | 25 | 22 | 22 | 22 | 23 | 24 | 24 | 24 | 22 | 24 | 24 | 24 | T |
| **Unique** | |  |  |  | 4 | 4 |  |  |  | 2 |  |  |  | 1 |  |  |  | U |
| **Monomorphic** | | 15 | 15 | 15 | 15 | 15 | 15 | 15 | 15 | 15 | 15 | 15 | 15 | 15 | 15 | 15 | 15 | M |
| **Polymorphic** | | 5 | 17 | 17 | 15 | 6 | 7 | 7 | 7 | 6 | 9 | 9 | 9 | 6 | 9 | 9 | 9 | P |
